# Ehrlichiosis presenting as severe sepsis and meningoencephalitis in an immunocompetent adult

**DOI:** 10.1099/jmmcr.0.005162

**Published:** 2018-07-27

**Authors:** Stephanie L. Buzzard, Brittany D. Bissell, Melissa L. Thompson Bastin

**Affiliations:** ^1^​ Department of Pharmacy Practice and Science, University of Kentucky College of Pharmacy, Lexington, KY, USA; ^2^​ Department of Pharmacy Services, University of Kentucky HealthCare, Lexington, KY, USA

**Keywords:** *Ehrlichia*, sepsis, meningoencephalitis, doxycycline

## Abstract

**Introduction:**

*Ehrlichia* are obligate intracellular pathogens transmitted to vertebrates by ticks.

**Case presentation:**

We report the case of a 59-year-old man who presented to the University of Kentucky Albert B. Chandler Medical Center (Lexington, KY, USA) after being found fallen down in the woods. A lumbar puncture revealed what appeared to be bacterial meningitis, yet cerebrospinal fluid cultures, Gram stains and a meningitis/encephalitis panel were inconclusive. However, an *Ehrlichia* DNA PCR of the blood resulted as being positive for *Ehrlichia chaffeensis* antibodies. The patient received a 14 day course of doxycycline, and recovered from his multiple organ failure. The aetiology of the ehrlichial meningoencephalitis was likely transmission through a tick-bite, due to the patient’s outdoor exposure.

**Conclusion:**

While it is rare to see *Ehrlichia* as a cause of meningitis, this illness can progress to severe multisystem disease with septic shock, meningoencephalitis or acute respiratory distress syndrome (ARDS). Those with compromised immunity are at a higher risk of developing the more severe form of the disease and have higher case fatality rates.

## Introduction

Ehrlichiosis is a bacterial illness that affects humans and animals causing flu-like symptoms. Symptoms commonly associated with this illness include fever, headache, fatigue and muscle aches. The majority of *Ehrlichia* transmission occurs during the summer months, peaking May through July. Since the early 2000s, cases of ehrlichiosis have steadily increased, and the estimated case fatality rate is 1–2 % [1]. Many individuals have mild symptoms and never seek medical attention. However, life-threatening cases of ehrlichiosis manifest as septic shock, meningoencephalitis or acute respiratory distress syndrome (ARDS). *Ehrlichia* spreads throughout the body via the mononuclear phagocytic system and may infiltrate the brain meninges, lung, kidney, gastrointestinal tract and heart [2]. In severe cases, patients are at an increased risk of death if not treated appropriately and in a timely manner [1, 2]. Despite low penetration into the cerebrospinal fluid (CSF), doxycycline is the treatment of choice for all patients with ehrlichiosis, and should be initiated as part of empiric therapy for patients living in an area where *Ehrlichia* is endemic [3]. Tick-borne meningoencephalitis is most common in adults, with increasing age being associated with less favourable outcomes [3].

## Case report

The following is a case presentation of a 59-year-old white male with a past medical history significant for depression and alcohol abuse, who was admitted to the emergency department at the University of Kentucky Albert B. Chandler Medical Center, Lexington, KY, USA, in spring 2017 with encephalopathy after being found fallen down in the woods. The patient was intubated prior to arrival due to altered mentation. Home medications included dexlansoprazole, venlafaxine, hydroxyzine, sulfasalazine, triamcinolone cream and amitriptyline. According to the patient’s pharmacy, he had no known medication allergies. The patient’s family was unable to provide an extensive past medical history, but indicated the patient lived alone in a cabin in the woods.

## Investigations

In the emergency department, the patient was febrile with a temperature of 103.5 °F (39.7 °C), a heart rate of 101 beats min^−1^ and blood pressure of 97/66 mmHg. An electrocardiogram was significant for a QRS interval of 104 ms, and a urine drug screen was positive for amitriptyline, venlafaxine and ketamine, which the patient received during intubation. Serum alcohol, acetaminophen and salicylate levels were undetectable. Given the mildly prolonged QRS interval and the presence of amitriptyline in the patient’s urine, he was given a 50 ml bolus of sodium bicarbonate 1 mEq ml^−1^ and started on a sodium bicarbonate infusion 0.15 mEq ml^−1^ due to concern for possible tricyclic antidepressant overdose. Other significant laboratory results included a white blood cell (WBC) count of 3.6×10^3^ cells µl^−1^ (normal range 3.7–10.3×10^3^ cells µl^−1^), a platelet count of 71×10^3^ platelets µl^−1^ (normal range 150–450×10^3^ platelets µl^−1^), international normalized ratio of 4.6, aspartate transaminase (AST) of 85 U l^−1^ (normal range 12–40 U l^−1^) and alanine aminotransferase (ALT) of 42 U l^−1^ (normal range 11–41 U l^−1^). A computed tomography (CT) scan of the head, abdomen and pelvis was normal; however, a CT scan of the chest was positive for basilar consolidations. Given the patient’s clinical presentation, meningitis was considered in the diagnostic differential. A lumbar puncture (LP) was performed, and the patient was started on acyclovir [600 mg intravenously (IV) every 8 h], ampicillin (2 g IV every 4 h), ceftriaxone (2 g IV every 12 h) and vancomycin [1250 mg (~13.4 mg kg^−1^) IV every 12 h] empirically. Blood, urine and sputum cultures, as well as meningitis/encephalitis panel PCR, were obtained from the patient prior to initiation of antibiotics. The organisms tested in the meningitis/encephalitis panel included: *Escherichia coli* K1, *Haemophilus influenzae*, *Listeria monocytogenes*, *Neisseria meningitidis*, *Streptococcus agalactiae*, *Streptococcus pneumoniae*, *Cryptococcus neoformans*/*Cryptococcus gattii*, cytomegalovirus, enterovirus, herpes simplex virus 1/2/6, human parechovirus and varicella zoster virus.

## Diagnosis

The following day, the results of the LP revealed a CSF WBC count of 28×10^3^ cells µl^−1^ (normal range 0–5×10^3^ cells µl^−1^), CSF glucose of 60 mg dl^−1^ (normal range 40–70 mg dl^−1^) and CSF protein of >600 mg dl^−1^ (normal range 15–45 mg dl^−1^). The LP results at this time were evident for bacterial meningitis. Due to the severity of the patient’s illness, and abnormally low WBC and platelet count, along with the history of living in the woods, intravenous doxycycline (100 mg twice daily) was started on day 2 of hospitalization for possible tick-borne disease. Prior to the administration of doxycycline, a serum DNA PCR test was sent to a reference lab, LabCorp Burlington NC, USA to test for *Anaplasma phagocytophilum* and *Ehrlichia chaffeensis* antibodies, both potential tick-borne illnesses most commonly seen in the south-eastern and south-central region of the USA. On day 3 of hospitalization, the patient had improvement in mental status, which was approximately 24 h after the initiation of doxycycline. The patient was awake and alert, and able to follow commands. At this time, the patient was successfully liberated from mechanical ventilation. The meningitis/encephalitis panel by PCR, along with the CSF culture, was negative. By day 5 of the patient’s hospital course, blood, urine and sputum cultures all revealed a final negative result. The infectious disease consult team recommended discontinuation of all antimicrobials except for doxycycline, as this patient fit an ehrlichial meningoencephalitis picture given the patient’s abnormal laboratory test values and abnormal LP results.

## Outcome and follow up

On hospital day 8, the DNA PCR resulted as negative for *Anaplasma phagocytophilum* antibodies and positive for *Ehrlichia chaffeensis* antibodies; the diagnosis of ehrlichial meningoencephalitis was confirmed. The results of this test took 6 days to return. By this time, the patient was afebrile with a temperature of 98.8 °F (37.1 °C) and continued to have rapid improvement in mental status. The patient’s WBC level had come up to 4.3×10^3^ cells l^−1^, his platelet count improved to 217×10^3^ platelets µl^−1^, the international normalized ratio had also normalized to 1.0. AST and ALT had come down to 52 and 36 U l^−1^, respectively. The patient was transferred to progressive care on day 10, and continued the doxycycline treatment for a 14 day course of therapy.

## Discussion

Human monocytic ehrlichiosis, caused by *Ehrlichia chaffeensis*, is commonly seen in the south-eastern and south-central USA, transmitted via the lone star tick [4]. This disease typically manifests as a flu-like illness, in which patients experience fever, headache, chills, malaise, nausea, vomiting and diarrhoea. However, when not treated appropriately, the disease may be fatal. These Gram-negative intracellular bacteria form as a microcolony in cytoplasmic vacuoles of monocytes and spread throughout the body via the mononuclear phagocytic system. *Ehrlichia* secrete proteins that pass through the vacuole membrane and bind to the host’s cytoplasmic proteins; thus, altering the host’s response to the infection by decreasing the host’s defence proteins such as IL-5, IL-18, chemokine receptors 2, 3 and 4, and MHC class II. While CD8^+^ T cells are thought to be immunoprotective, they also contribute to the lethal immunopathology of ehrlichiosis. CD8^+^ T cells overproduce TNF-α, leading to the induction of apoptosis of hepatocytes [5]. High levels of serum TNF-α are associated with severe and fatal disease, which can present as a septic shock and multiple organ failure [6].

Neurological manifestations of ehrlichiosis in humans are rarely reported in the literature. A PubMed (1959–2018) search was limited to the English language and humans using the keys words ehrlichiosis AND meningoencephalitis and revealed four case reports of CNS manifestations of ehrlichiosis, which are summarized in Table 1 [7–10].

**Table 1.** Reports of CNS manifestation of *Ehrlichia* in the medical literature

**Patient**	**Presentation**	**Temperature**	**Microbiology**	**CSF results**	**Other pertinent findings**	**Outcome/treatment**
Our case	Found unconscious, encephalopathic, acutely agitated	103.5 °F	(−) CSF culture (+) *Ehrlichia* DNA PCR	Protein >600 g dl^−1^ WBC 28 k µl^−1^	WBC 3.6 k µl^−1^ Platelets 71 k µl^−1^ AST 85 U l^−1^ ALT 42 U l^−1^	Diagnosed with ehrlichial meningoencephalitis. Treated with doxycycline 100 mg twice daily for 14 days, and discharged to floor on day 10.
45 male [7]	Found unconscious and unresponsive, presented in status epilepticus with reddish-purple petechiae	102.3 °F	(+) *Ehrlichia* DNA PCR	Not applicable	Platelets 6 k µl^−1^ AST 750 U l^−1^ ALT 117 U l^−1^	Meningoencephalitis with evidence of infection with *Ehrlichia chaffeensis*. Patient rapidly deteriorated and died before intervention could be made.
45 male [8]	Acute onset of nonpositional bilateral retro-orbital headache accompanied by nausea, emesis and photophobia	103 °F	(+) CSF *Ehrlichia* DNA PCR	Red blood cell count 1593 M µl^−1^ WBC 57 k µl^−1^ Protein 101 g dl^−1^	Platelets 86 k µl^−1^ AST 177 U l^−1^ ALT 229 U l^−1^	Diagnosed with *Ehrlichia chaffeensis* infection and treated with doxycycline 100 mg BID for 14 days. Patient made a complete recovery.
47 male [9]	Severe headaches, photophobia, body aches and pain	102 °F	(−) CSF culture (+) CSF and blood *Ehrlichia* DNA PCR	WBC 19 k µl^−1^ Protein 63 g dl^−1^	AST 111 U l^−1^ ALT 113 U l^−1^	Diagnosed with aseptic meningitis/*Ehrlichia chaffeensis.* Showed initial improvement with doxycycline; however, did not complete course of therapy.
38 male [10]	Nausea, vomiting, headache, rash and thrombocytopenia	Not applicable	CSF IgG *Ehrlichia* titre 1 : 1280 (admission); 1 : 320 (4 weeks later)	WBC 117 k µl^−1^	WBC 5.9 k µl^−1^ Platelets 51 k µl^−1^	Diagnosed with Lyme and ehrlichia meningitis, promptly started on doxycycline, discharged home and did well at 4 week follow up.

The species of *Ehrlichia* in our patient’s case was *Ehrlichia chaffeensis*, a species commonly found in the south-eastern and south-central USA [1]. This was confirmed by laboratory testing by LabCorp Burlington. If delayed identification leads to delayed treatment, this can worsen outcomes for these patients. A study by Hamburg *et al*. showed that patients who had a delay in the initiation of doxycycline for ehrlichiosis infection had an increased rate of transfer to the intensive care unit and requirement for mechanical ventilation, as well as a longer hospital stay and longer length of illness [11]. Unfortunately, treatment delays are likely due to the lack of recognition of the diagnosis of ehrlichiosis [11]. These data suggest that in areas where *Ehrlichia* infection is endemic, providers should have a heightened awareness and a lower threshold for initiating doxycycline therapy in patients with a suspicion of ehrlichiosis. A thorough patient history, including previous travels, occupation and leisurely activity, is important.

Overall, this case is noteworthy due to the low prevalence of ehrlichial meningoencephalitis and difficulty achieving a rapid diagnosis. Meningoencephalitis is a complication secondary to severe ehrlichiosis, rarely reported in the medical literature. Patients most at risk for severe forms of ehrlichiosis are usually immunocompromised. While this patient had risk factors for ehrlichiosis, including older age and residing in the woods, he was not otherwise immunosuppressed.
